# A Low-Code Framework for Complex Crowdsourcing Work Based on Process Modeling

**DOI:** 10.1155/2022/9496741

**Published:** 2022-04-29

**Authors:** Tianhong Xiong, Maolin Pan, Yang Yu, Dingjun Lou

**Affiliations:** School of Computer Science and Engineering, Sun Yat-sen University, Guangzhou 510006, China

## Abstract

Crowdsourcing has become a new distributed paradigm, which uses online crowds to solve complex problems. Recently, in order to reduce the development workload and research threshold of crowdsourcing applications, crowdsourcing process modeling is attracting more and more attention. However, complex crowdsourcing processes used for creative and open-ended work have remained out of reach for process modeling, because this type of process usually has a dynamic execution, in which the type, number, and order of tasks and subtasks are often unknown in advance but are determined dynamically at runtime. In this paper, we propose a modeling approach and supporting framework to fill this gap. Specifically, we provide a task model composition to allow task creation on demand, while collaborating on tasks in a tree structure to adapt to the dynamic execution. Moreover, we introduce a set of message communication modes to support data exchange among tasks. Finally, we construct a framework named CrowdModeller to embody this approach. Through two evaluations, we demonstrate its effectiveness.

## 1. Introduction

Crowdsourcing is becoming a new online business model that allows *requesters* (employers) and crowdsourcing *workers* (employees) to solve problems through network interaction and collaboration [1]. Many problems are solved as a series of simple tasks (e.g., microtasks) [2], such as labeling an image, checking the accuracy of a set of data, and processing a natural language text segment. Furthermore, to complete more complex crowdsourcing work (e.g., creative software design and article writing), the requester usually needs to link multiple tasks to a *crowdsourcing process* [3]. Such a process, regarded as a distributed paradigm, can decompose large and complex tasks into small and simple subtasks and then recombine subtask solutions into a whole work product [4]. In this context, it is important to provide effective support for crowdsourcing process design and implementation.

In early work, a number of crowdsourcing toolkits have been created to *programmatically* support crowdsourcing processes. These toolkits are mostly oriented to specific programming languages (e.g., JavaScript [5, 6], Dog [7], Scala [8], and Python [9–11]), helping requesters to quickly customize crowdsourcing processes. Unfortunately, this kind of toolkit is not friendly enough for requesters, especially those without specific programming background and skills, so that they either rely on professional developers or have to master specific programming methods.

Currently, many studies (e.g., [12–18]) try to provide fast and convenient solutions for crowdsourcing process with the help of low-code technology [19], especially employing business process modeling [20] to reduce the dependence on programming skills. These modeling approaches provide graphical modeling tools to visualize the design, implementation, and maintenance of crowdsourcing processes. They enable requesters to focus more on the formulation and improvement of crowdsourcing processes (design and adjustment of process models), rather than software development (large amount of program hard coding).

However, the above crowdsourcing modeling approaches mainly focus on the static control flow perspective that predefines all tasks in a single fixed model and coordinates tasks in an assembly line-like structure. Less attention has been paid to formal modeling that can directly support complex crowdsourcing work (i.e., creative and open-ended work). In fact, supporting more creative and valuable open-ended work is becoming a research trend in crowdsourcing community [21]. This type of work has a wide range of applications, e.g., article writing, software design, and creative design, attracting more and more interest [22]. Its key feature is that there is often no clearly defined solution, allowing workers to participate in task partition or self-coordination [21]. Thus, the crowdsourcing process for such work usually has a *dynamic execution*, in which the type, number, and order of tasks and subtasks are often unknown in advance but are determined dynamically by workers at runtime. For example, the requester may not be able to specify all sections of an article beforehand but allows workers to participate in task partition according to a brief outline. In particular, when a section task in an article writing case is unclear or too complex at runtime, it is further refined and decomposed into sub-section tasks until they are executable. As a result, how to model, coordinate, and manage tasks to adapt to this dynamic execution is a key challenge for crowdsourcing process modeling.

In this paper, we fill this gap by providing a formal modeling approach and a web-based support framework. Its core is to construct a task model composition to allow task creation and data exchange on demand, while managing tasks (instances) in a tree structure to adapt to the dynamic execution. Specifically, a crowdsourcing process is characterized by a *task model composition* containing multiple task models. The task model describes the state change of task from beginning to end, driving task partition, execution, and routing. Meanwhile, we introduce *instance tree* to facilitate the task collaboration and management. The instance tree is not static but is dynamically generated during runtime, in which the nodes represent tasks created by task models and the arrows represent task dependencies (parent-child creation relationships). Furthermore, we provide a set of *message communication modes* to support task coordination, so that the data exchange can be performed accurately among tasks. Tasks do not exist in isolation but are managed by instance tree to meet the needs of dynamic execution.

This paper extends our previous work [23] in the following five aspects: we introduced the model composition to represent the crowdsourcing process, rather than a single specific model; we gave the formal definition of task model composition and instance tree, rather than the text description of a single model; we improved the framework structure, model design, and implementation interfaces; we provided a more comprehensive comparison of the existing crowdsourcing modeling approaches; and we conducted a set of more extensive experiments.

In summary, we make the following main contributions in this paper:

1. A crowdsourcing process modeling approach, which combines task model composition, instance tree, and message communication modes to handle task modeling, collaboration, and data exchange.
2. An automated execution framework named CrowdModeller, which enables the requesters to graphically model (design), implement, and manage crowdsourcing processes.
3. Two evaluations, which show that CrowdModeller can accommodate the needs of complex crowdsourcing work, especially creative and open-ended work.

The rest of this paper is organized as follows.  Section 2 gives an overview of related work.  Section 3 describes the approach and execution framework in detail.  Section 4 reports two evaluations based on article writing example.  Section 5 discusses the strengths and shortcomings of the approach.  Section 6 summarizes the paper and suggests future work.

## 2. Related Work

In this section, we firstly summarize the existing crowdsourcing approaches from multiple dimensions, including process definition language, control flow, data management, as shown in Table 1. Then, we take the “process definition language” as the benchmark to classify existing work into two categories, *language-specific programming* and *process modeling* (low code), and analyze them separately. Finally, we discuss the latest research on the complex and open-ended work, which is also closely related to our work.

Both language-specific programming [5–11] and process modeling [12–18,24] support crowdsourcing processes through different strategies. The former integrates program scripts, task templates, and advanced algorithms to facilitate rapid *process development*, while the latter employs custom modeling and extended standard modeling languages (e.g., BPEL and BPMN), to enable requesters to focus more on visual design and management of crowdsourcing processes, rather than heavy program coding. With respect to *control flow mechanism* and *data management*, both of these approaches can provide good support in crowdsourcing processes. However, existing process modeling mainly focuses on static control flow when considering *process execution*, i.e., prespecified task partition and fixed execution, so that it is hard to provide support for dynamic execution.

### 2.1. Language-Specific Programming

Early work has proposed many language-specific programming approaches. TurKit [6] provides a JavaScript-oriented programming toolkit to quickly deploy tasks in an iterative manner. Soylent [5] introduces “find-fix-verify” crowd programming paradigm to divide and execute tasks on the basis of TurKit, so as to improve the quality of text editing. Jabberwocky [7] develops a high-level programming language called Dog and uses it to support cross-platform crowdsourcing processes. Moreover, AutoMan [8] is a crowdsourcing programming framework that integrates human-based and digital computing. It uses Scala language and algorithms based on quality control, scheduling, and budget to automate process implementation. Different from the above, CrowdForge [9] and Turkomatic [10] both adopt a divide-and-conquer strategy to solve crowdsourcing problems. The former proposes a multilevel partition structure based on MapReduce paradigm and combines Python and task template to create tasks, while the latter introduces price-divide-solve algorithm based on task price to achieve crowdsourcing goals. In addition, Cascade [11] uses an iterative algorithm to solve the classification work, which supports parallel task execution. The above approaches enable the requesters to customize and deploy crowdsourcing applications programmatically. Unfortunately, they are not friendly enough for requesters, such as business experts and analysts, who often lack specialized programming background and skills [18, 24].

### 2.2. Process Modeling

In order to further reduce the development workload, as well as the dependence on programming skills, many studies attempt to evolve from programming approaches to crowdsourcing process modeling. As one of the classic low-code solutions, the process modeling provides a graphical way to quickly implement crowdsourcing process. In earlier work, CrowdLang [12] describes a process-driven modeling language. It implements crowdsourcing processes by customizing a set of operators, including Reduce, Aggregate, and Multiply. CrowdWeaver [13] provides a graphical modeling tool to configure crowdsourcing tasks, including human and machine tasks, and to manage data flows visually.

Recently, the main work of [17] is to employ the BPEL4People, an extension of the web service orchestration language BPEL, to model and implement crowdsourcing processes. Unlike our work, it focuses on assigning tasks to appropriate workers according to nonfunctional attributes, such as worker's ability and skill level. CrowdSearcher [14] introduces the concept of modeling and defines a set of task types (such as Tag, Like, Order, Classify, and Group) and patterns to organize crowdsourcing processes. Unfortunately, its modeling notations and semantics are customized, not from the standard modeling languages. To fill this gap, Crowd Computer [18] extends the standard BPMN to customize crowdsourcing tactics, including contest and auction. It uses an open source execution engine to separate business process logic from implementation logic and supports multiple control flow mechanisms to implement tasks. Overall, it focuses on modeling and integration of static crowdsourcing processes without considering open-ended work that has no clearly defined solution. Similar work can be found in [15, 16]. They provide a web-based framework to support hybrid human-computer computing, emphasizing task scheduling. Furthermore, Service4Crowd [24] introduces a service-oriented crowdsourcing platform to develop and manage service compositions. Each service represents an activity (task) of the crowdsourcing process, which is parsed and scheduled by an open source BPMN engine. Although its service composition can flexibly meet the definition and execution of the crowdsourcing tasks, it is still limited to a single fixed process model and does not consider the dynamic execution.

### 2.3. The Researches on Complex and Open-Ended Work

Another noteworthy related work is the researches and discussions on complex and open-ended work, which inspired our work. Kittur [21] discusses complex crowdsourcing processes with the help of organizational behavior and distributed computing. The researcher points out that the open-ended work consists of interdependent tasks that require continuous collaboration (dynamic execution) to achieve business goals. In particular, there are a number of interdependencies that need to be addressed, including shared human resources, input/output data dependencies, and task/subtask dependencies. Furthermore, Retelny et al. [25] argue that the current crowdsourcing approaches focus mainly on static execution process and lack the necessary adaptability to changing requirements, such as upstream and downstream task collaboration and changing goals. These characteristics pose new challenges to the crowdsourcing process modeling. Similarly, Vaish et al. [26] analyze task dependencies based on data view, indicating that complex work may achieve global goals through a large number of local (individual) efforts. Inspired by these, we explore combining business process modeling techniques to cover the dynamic execution features of complex crowdsourcing processes, especially when considering open-ended work.

## 3. CrowdModeller

In this section, we first introduce the modeling approach and then describe the corresponding support framework named CrowdModeller.

### 3.1. Modeling Approach

**Figure 1:**
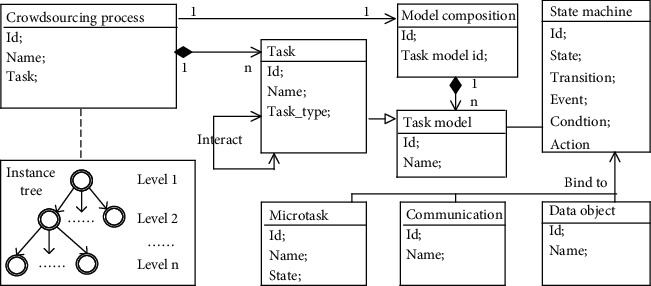
Metamodel of the approach.

To accommodate the features of complex crowdsourcing processes, our approach mainly considers three aspects, i.e., task modeling, task collaboration, and data exchange.  Figure 1 shows the metamodel of the approach. A crowdsourcing process is associated with a task model composition, in which each task model is represented using a state machine. The state machine not only represents the state change of task, but also binds several service interfaces to perform specific operations, e.g., publishing microtasks, enabling communication, and storing data. It especially supports invoking any model that belongs to the model composition to create subtasks, including itself or other task models. At runtime, each task is instantiated by invoking the task model from the composition, while tasks can interact with each other through message communication modes. Furthermore, all tasks gradually form an instance tree, in which the nodes represent tasks created by task models and the arrows represent task dependencies (parent-child creation relationships).

Level 1 and level 2 represent different layers of the instance tree. Usually, the instance in level 1 is created first, and then the instance of level 2 is triggered by the instance of level 1. In short, the *model composition* represents the static perspective of crowdsourcing process, while the *instance tree* shows the dynamic execution of crowdsourcing process. They work together with *message communication modes* to achieve task modeling, coordination, and data exchange.

#### 3.1.1. Task Model

**Figure 2:**
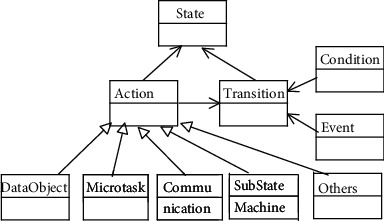
The class diagram of extended state machine.

The state machine is extended to describe task model, where *action* encapsulates a set of service interfaces to support data manipulation and external interactions, as shown in Figure 2. Formally, the task model is defined as follows.

**Definition 1 .**

(task model, TM). A task model is an 8-tuple based on state machine *TM* (*S*, *s0*, *F*, *C*, *E*, *A*, *L*, *T*), where the following notations are used:

1. *S* is a finite set of task *states*, *s0* ∈ *S* is the initial state, and *F* ⊆ *S* is the set of final states.
2. *C* is a set of *Boolean conditions*: *T* (*true*) and *F* (*false*) ∈ *C.* Let *Ci* be a set of primitive conditions; if *c* ∈ *Ci*, then *c* ∈ *C*; if *c1* ∈ *Ci*, *c2* ∈ *Ci*, op ∈ { =,<,>,≠,≤,≥}, then *c1* op *c2* ∈ *C*; *c1* ∨ *c2*, *c1* ∧ *c2*, and ∼ *c1* ∈ *C*.
3. *E* is a set of *events.* If *c* ∈ *C* and *e* ∈ *E,* then *e*[*c*] ∈ *E*.
4. *A* is a set of *actions.* If *vx* ∈ *V*, then *v1*: = *v2* ∈ *A*, and *V* is a set of expressions. Besides expressions, actions are bound to a set of interface templates *it* (*type*, *template*), where *type* denotes the types of interfaces, and *template* is a series interface parameters.
5. *L* ⊆ *E* × *C* × *A* is a set of *labels.* It is written as *e*[*c*]/*a*, where *e* ∈ *E*, *c* ∈ *C*, *a* ∈ *A*.
6. *T* ⊆ *2*^*S*^ × *L* × *2*^*S*^ is a set of *transitions.* For a transition *t* = (*s1*, *l*, *s2*), *s1* is the source state and *s2* is the target state; *l* ∈ *L* is a label. A transition *t* is triggered if *s1* is enabled and *e* ∈ *E* is activated with *c* ∈ *C* being true, causing *a* ∈ *A* to be taken.

In Definition 1, (1) defines the execution states of the task model. (2), (3), and (4) together form a set of labels (i.e., (5)), driving the migration of task state. When a transition in (6) is triggered, the task state transfers from the source state to the target state. In particular, (4) provides a set of interface templates through actions, including the following: *DataObject* is used to read/write data; *Microtask* is responsible for publishing a simple microtask; *Communication* allows invoking a message communication mode to send messages to other tasks; *SubStateMachine* invokes a task model to create a new task instance, as shown in Figure 2.

**Definition 2 .**

(task model composition, TMC). Let *TM*_*1*_, *TM*_*2*_,…, *TM*_*n*_ be a set of task models that participate in the crowdsourcing process. A *TMC* is defined as *TMC* = *TM*_*1*_||…||*TM*_*n*_, where || is a parallel operator.

Note that Definition 2 is only a structure definition that defines a parallel relationship between task models, while the coordination and interaction between *TM*_*i*_ rely on message communication modes (see Definition 4). The task models in the model composition can establish a variety of coordination and interaction mechanisms with each other by combining the communication modes and instance tree. In this way, it can adapt to the dynamic execution in complex crowdsourcing process.

#### 3.1.2. Instance Tree

**Figure 3:**
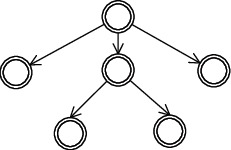
A form of instance tree. Each node represents a task created by a task model.

The instance tree is introduced to coordinate and manage tasks during dynamic execution. A form of the instance tree is shown in Figure 3.

**Definition 3 .**

(instance tree, IR). An instance tree is a 2-tuple *IR* (*N*, *R*), where *N* is a set of nodes created by task model *TM*_*i*_, where *TM*_*i*_ ∈ *TMC*, *1* ≤ *i* ≤ *n*.

1. *R* ⊆ *N* × *N* is the set of edges (parent-child creation relationships) formed by the ordered pairs (*np*, *nc*), where *np* as the parent node calls a task model to create its subtask *nc*.
2. *nroot* ∈ *N*, ∀ (*n*, *nroot*) ∈ *R*: *nroot* is a root.
3. *nleaf* ∈ *N*, ∀ (*nleaf*, *n*) ∈ *R*: *nleaf* is a leaf.

It should be noted that the instance tree is not fixed because it is generated dynamically at runtime; that is, dynamic execution may result in different forms of instance tree. As a result, we can observe that *TMs* and *TMC* are static models that are designed by the requesters, while *IR* describes dynamic execution under *TMC* constraints.

#### 3.1.3. Message Communication Mode

**Figure 4:**
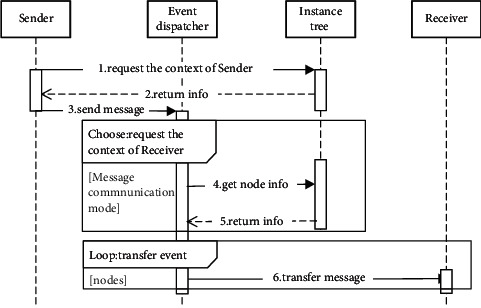
Sequence diagram of message communication modes. First, the sender obtains its own context (e.g., ID, state and location) from the instance tree as a preset condition. Then, the event dispatcher obtains the context of the receiver according to the sender instruction and the selected message communication mode. Finally, it passes the message to the corresponding receiver.

A common set of message communication modes are provided to facilitate collaboration and data exchange between tasks. To ensure that the message is delivered to the receiver accurately, the message communication modes rely on the instance tree to provide the task context, e.g., task id, state, and location. In more detail, we provide a sequence diagram to show how the message communication modes work (see Figure 4 in Section 3.2).

**Figure 5:**
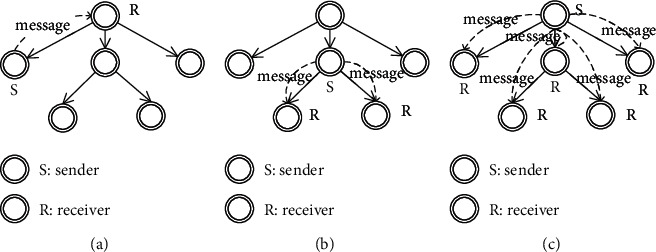
Three types of message communication modes based on instance tree. (a) ToParent. (b) ToChild. (c) ToDescendant.

Tree structure itself is a simple and flexible mechanism. In theory, communication can be established between any nodes based on instance tree. In practice, we provide three common message communication modes: ToParent, ToChild, and ToDescendant, as shown in Figure 5. If necessary, more modes can be obtained based on the instance tree. Note that in order to simplify the definition and clearly show the message interaction between task models, we assume that the message name is unique.

**Definition 4 .**

(ToParent message communication mode). ToParent means that a child task sends a message to its parent task. Formally, *ToParent* = (*n*_*i*_ × *m* × *n*_*j*_), where (*n*_*j*_, *n*_*i*_) ⊆ *R*, *n*_*i*_ ∈ *N* represents a sender in state *i*, *m* ∈ *M* represents a message, and *n*_*j*_ ∈ *N* represents a receiver in state *j*.

The ToParent is a common point-to-point mode that has only one receiver and sends only one message at a time. It is often used by subtasks to return results to the parent task.

**Definition 5 .**

(ToChild message communication mode). ToChild means that a parent task sends messages to its child tasks. Formally, *ToChild* = (*n*_*i*_ × *m* × *n*_*j*_), where (*n*_*i*_, *n*_*j*_) ⊆ *R*, *n*_*i*_ ∈ *N* represents a sender in state *i*, *m* ⊆ *M* is a set of messages, and *n*_*j*_ ⊆ *N* represents a set of receivers in state *j*.

The ToChild allows the sender to send messages to its child tasks. It is often used by the parent task to send task information to the child tasks.

**Definition 6 .**

(ToDescendant message communication mode). ToDescendant means that a sender sends messages to all its descendants. Formally, *ToDescendant* = (*n*_*i*_ × *m* × *n*_*j*_), where *n*_*i*_ ∈ *N* represents a sender in state *i*, *m* ⊆ *M* is a set of messages, and *n*_*j*_ ⊆ *N* is a set of descendant nodes of *n*_*i*_ in state *j*.

The ToDescendant is an extension of ToChild. When applied to the root node, it affects the entire instance tree. For example, before the end, the process will use this mode in the root node to send messages to all descendant tasks to query whether they are completed.

### 3.2. Implementation

At present, state machine and its variants have become mature graphical modeling tools for describing complex systems [27]. More importantly, the World Wide Web Consortium (W3C) combines the semantics of statechart [28] (a variant of state machine) with XML grammar to propose a general state machine language called SCXML. It has normative executable semantics and has received positive response and support from academia and industry [29, 30]. In particular, Apache Commons project (http://www.apache.org) has developed an open source engine called Commons SCXML to parse and execute state machine models defined using SCXML, providing rich advanced APIs to extend custom operations and semantics. These existing foundations facilitate the implementation of modeling approach and framework in this paper.

**Figure 6:**
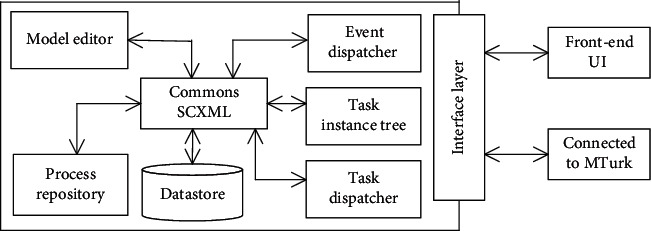
High-level architecture of CrowdModeller. Two-way arrows indicate that these components can interact with each other. The source code of the framework has been uploaded to GitHub (https://github.com/xthHub/RenWFMS).

We build an automated execution framework, CrowdModeller, to embody the modeling approach.  Figure 6 shows its high-level architecture.

**Figure 7:**
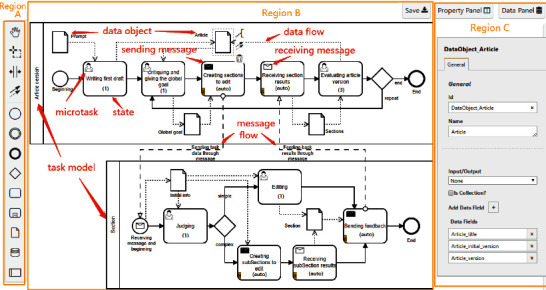
The model editor in CrowdModeller. Region B is a modeling whiteboard, while Region A is the Model Notation Panel. Region C contains the Property Panel and Data Panel.

*Model editor* is a web-based graphical design tool that allows the requester to design task models and associate them to a model composition.  Figure 7 shows a TMC of an article writing case that will be used in Section 4. Region B is a modeling whiteboard where a model composition with two task models has been constructed, i.e., article version and section task model. The dotted arrows inside the models represent the data flow, while the dashed line hollow arrows between the two models represent the message flow. The black envelope indicates that the state can invoke message communication modes to send messages, while the white envelope indicates that the state can receive messages sent by the external model. A human shape indicates that the state can publish microtasks, and the number in bracket indicates the number of microtasks. Region A is the Model Notation Panel. The Property Panel and Data Panel in Region C are used to configure task properties and data object parameters in model composition.

*Commons SCXML* is a Java-based open source workflow engine for performing task model composition defined by the model editor. Specifically, the engine parses the model into the *process repository* and *Datastore* and drives the task execution. *Event dispatcher* works with the *instance tree* to pass messages between tasks, as shown in Figure 4. The *task dispatcher* is responsible for creating and scheduling tasks. The *interface layer* provides a set of service interfaces connected to external services. In particular, it integrates ParlAI [31] for publishing microtasks to other crowdsourcing markets, e.g., MTurk (https://www.mturk.com), and manages data interaction with the front-end UI.

## 4. Evaluation

In this section, we conduct two evaluations to illustrate the effectiveness of CrowdModeller. The first evaluation, as a benchmark study, uses an article writing case to illustrate how CrowdModeller coordinates task models, instance tree, and message communication modes to efficiently adapt the complex crowdsourcing processes. The second evaluation, as a comparative study, compares CrowdModeller with the latest crowdsourcing process modeling approach based on the same experimental conditions. A group of 20 graduate students were invited to participate in the experiment as crowdsourcing workers. They were free to choose microtasks and complete them according to the prompts.

### 4.1. Benchmark Study

The selected article writing case is derived from the literature [22], whose goal is to obtain a high-quality article based on a brief prompt provided by the requester. The reason for choosing article writing is that it is a typical open-ended work without a well-defined solution [21]. Although this case has been explored programmatically, it is still out of reach for crowdsourcing process modeling approaches.

Specifically, the article writing case obtains articles by constantly updating the global goal and executing the local goals with workers, rather than simply requiring workers to execute a static goal. The global goal represents the writing direction and requirements of the whole article, while the local goals are decomposed from the global goal, corresponding to the sections of an article. In particular, due to the complexity of requirements, each section may be further decomposed into subsections at runtime until they are executable.

In order to adapt to the dynamic execution, especially the uncertainty of section tasks, we design a model composition, which contains two task models (i.e., article version task model (AVTM) and section task model (STM)) to achieve global and local goals respectively, as shown in Region B of Figure 7. Note that a human shape indicates that the state can post microtasks to workers, the number in bracket indicates the number of microtasks, and the “(auto)” means that it is an automatic operation.

The AVTM is responsible for managing the whole writing process, including choosing the global goal, decomposing the goal into specific sections, receiving the results of the section tasks, and evaluating the new article version. On the other hand, the SAM is in charge of section editing. In particular, it contains data-based routing to assist the worker decides whether the current section needs further decomposition. Moreover, the task models also have the following main characteristics.

AVTM and SAM interact and exchange data through message communication modes. In state *creating sections to edit*, the AVTM enables actions *SubStateMachine* and *Communication*, that is, it sets up SAM to create section tasks and *ToChild* mode to interact with the section tasks it creates. Meanwhile, it receives the feedback in the next state (*receiving section results*). Accordingly, SAM has a corresponding state to receive messages or send feedback messages; e.g., in state *sending feedback*, it sets up *ToParent* mode to return the feedback to its parent task.

Note that tasks belonging to SAM can also interact and exchange data with each other. In state *creating subSections to edit*, it enables actions *SubStateMachine* and *Communication* too. That is, it calls SAM to create sub-section tasks and *ToChild* mode to interact with its sub-section tasks.

#### 4.1.1. Experimentation Results and Analysis

We use two snapshots to illustrate the experiment process and give an analysis and summary.

**Figure 8:**
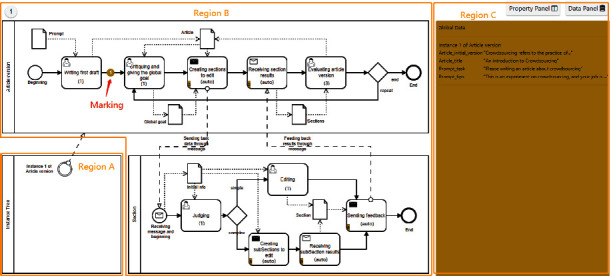
An execution snapshot with only one article version task currently running.

Initially, we give a brief prompt, i.e., “please write an article about crowdsourcing,” as input to the experiment. In state *writing first draft*, a worker is asked to complete the first draft based on this prompt. After that, Figure 8 shows the current execution snapshot. Region A at the lower left represents the instance tree, showing that only one article version task (for short AVTM task) is currently running. Region B displays the execution state of the task in real time. At this point, a circular *marking 1* represents the AVTM task 1, indicating that it is moving from the state *writing first draft* to the next state. During execution, each task is represented by a specific *marking*. Region C shows the main runtime data of task 1, including the first draft and title.

**Figure 9:**
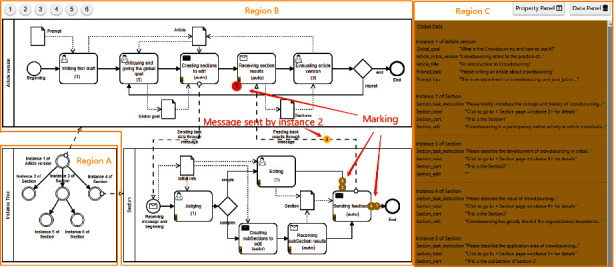
An execution snapshot showing that an article version task and five section tasks are running, while section task 3 is decomposed into two sub-section tasks 5 and 6.

Figure 9 provides an execution snapshot showing that an article version task and multiple section tasks (SAM) are running. Region A shows the real-time situation of the instance tree. Compared with Figure 8, it adds multiple task instances. Specifically, the AVTM task 1 creates SAM tasks 2, 3, and 4, while task 3 continues to be decomposed into sub-SAM tasks 5 and 6. Region B shows that AVTM task 1 (red *marking 1*) is waiting for feedback from other tasks in state *receiving section results*. On the one hand, SAM tasks 2 and 4 (yellow *markings 2 and 4*) are entering the *end* state, and they are feeding back the results to the AVTM task 1 through messages (a pale yellow *marking 2* indicates the message sent by task 2). On the other hand, SAM tasks 5 and 6 (yellow *markings 5 and 6*) are entering the *sending feedback* state, indicating that they are ready to feed back the results to their parent task (i.e., task 3). Region C displays the runtime data for each task.

**Figure 10:**
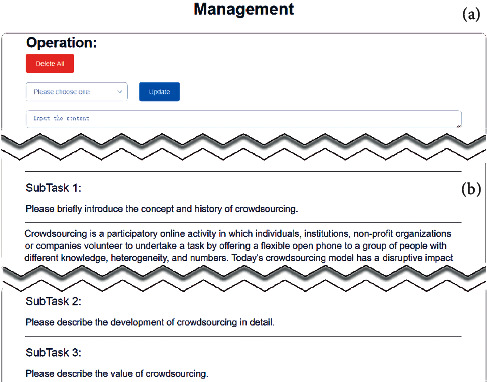
Management interface. (a) Basic operations to adjust task instructions, such as modifying task descriptions or deleting tasks when the quality of tasks is too low or for other reasons. (b) Detailed instructions and results of tasks.

Here, limited to the interface layout, Region C only shows the main task data, while more detailed information including microtask data is displayed in the built-in front-end interfaces, as shown in Figure 10. The final outputs are shown in the appendix.

This evaluation shows that CrowdModeller integrates task model composition, instance tree, and message communication modes to effectively adapt to the complex crowdsourcing processes. Specifically, it introduces two task models to constitute a model composition, adapting to different levels of goals in the article writing case. The tasks can flexibly invoke the other model or their own model to create subtasks during execution. Moreover, CrowdModeller establishes a “communication bridge” between tasks through instance tree and message communication modes. Finally, it clearly shows the dynamic execution of the whole crowdsourcing process, helping the requester to intuitively monitor and understand the crowdsourcing process. In summary, CrowdModeller can visualize the design, execution, and management of the entire crowdsourcing process, supporting complex task modeling, collaboration, and data exchange requirements for dynamic execution.

### 4.2. Comparative Study

In this evaluation, we use the same article writing case to compare CrowdModeller with the latest process modeling approach, namely, Service4Crowd. Specifically, based on the same experimental conditions, we conduct a two-round comparative experiment.

**Figure 11:**
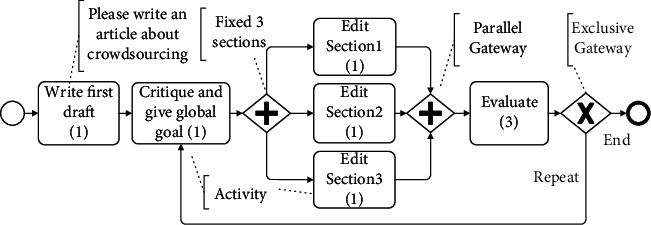
The article writing model based on Service4Crowd.

Service4Crowd provides visual modeling based on BPMN to design the crowdsourcing process, as shown in Figure 11. It is a single fixed model that predefines all tasks, including setting a global goal, breaking down sections, and editing sections. The requester uses graphical symbols to describe the process model of the whole article writing, such as writing a first draft and setting a global goal. In particular, the model contains a pair of parallel gateways to divide the article into three sections. All section tasks are linked together in a linear structure because all tasks are specified in advance by the requester. Furthermore, since the number of sections is fixed and cannot be modified during execution, when entering the second round, the new version of the article is still divided into three sections to be revised, regardless of the quality of the first round. As a result, this may cause it to lack the necessary support in the face of changing requirements (dynamic execution). For example, in the second round, some sections need no modification, but modifications are enforced, while some sections need to be further decomposed, but this cannot be implemented.

Instead, CrowdModeller provides a model composition to coordinate multiple task models (including AVTM and SAM) to collaborate and interact. In the first round, one AVTM task and five SAM tasks are created (see Figure 9). Especially in the second round, the worker evaluates the article obtained from the first round and chooses the new global goal: the entire article (5 sections) does not need to be fully revised; only three sections need to be revised. As a result, three new section tasks are created in the second round, instead of the same five section tasks as in the first round. Detailed comparison statistics are shown in Table 2.

The experimental results show that although both CrowdModeller and Service4Crowd can design the article writing process by visual modeling, the former is better able to adapt to changing requirements than the latter. That is, the way of model composition provided by CrowdModeller is more flexible in dynamic execution than a single fixed model.

## 5. Discussion

### 5.1. Comparative Analysis

**Figure 12:**
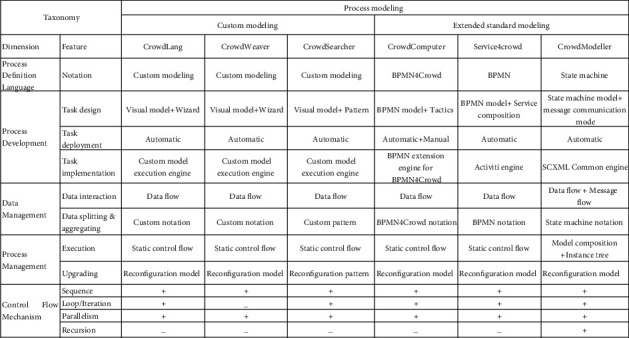
Comparing CrowdModeller with other modeling approaches from multiple dimensions, especially in task design, data interaction, and process execution.

To further illustrate the strengths and shortcomings of CrowdModeller, we qualitatively compare it with other process modeling approaches from multiple dimensions. This is a further refinement based on Table 1. The reasons for choosing these approaches are as follows: On the one hand, available source code or online resources can provide sufficient information for analysis. On the other hand, they represent the state of the art for crowdsourcing process modeling. According to the dimension of *process definition language*, we divide the existing process modeling approaches into two categories: custom modeling and extended standard modeling, as shown in Figure 12.

At present, when considering *process definition language*, many researches have gradually evolved from *custom modeling* to *extended standard modeling*, because the widely used standard modeling specifications can provide more theoretical and practical support, facilitating the design and implementation of crowdsourcing processes, such as in *process development*, *data management*, and *process management*.

Specifically, in *process development*, CrowdModeller develops crowdsourcing process in the way of task model composition, which creates tasks on demand in dynamic execution. Task models collaborate and interact with each other through message communication modes. The requesters can customize model constructs and configurations through graphical state machine symbols and rich service interfaces, including specifying microtasks and setting up automated tasks handled by the system (e.g., calling task models to create task instances and sending/receiving messages). Moreover, the task models conform to the standard SCXML specification and have a solid theory and application foundation compared with the *custom modeling*. In particular, CrowdModeller supports models with arbitrary topologies and has no inherent limitations similar to those in other approaches, such as specific crowdsourcing patterns in CrowdSearcher and crowdsourcing tactics in Crowd Computer.

With respect *to data management*, CrowdModeller achieves data exchange by combining data flow (object) and message flow. In particular, based on instance tree, it provides a set of message communication modes to support sending and receiving messages, so that the data can be transmitted accurately between tasks. The execution data of each task, such as task status, task data, and relationship between tasks, can be shown in CrowdModeller (Figures 8 and 9). Note that more detailed microtask data is shown on the built-in front-end UIs (Figure 10). These features enable the requester to monitor and manage data in real time during execution.

In *process management*, CrowdModeller provides model composition to model crowdsourcing process statically and introduces instance tree in dynamic execution. The former allows the requester to flexibly configure the model, while the latter clearly shows the running task context.

### 5.2. Limitations

We observe some shortcomings in CrowdModeller in the evaluations. On the one hand, for some specific crowdsourcing work, such as classification and labeling [9], CrowdModeller has no special advantages over other approaches, with even more complexity; because this type of work is usually non-open-ended, the design and implementation of task model composition may lead to unnecessary complexity. From this point of view, CrowdModeller focuses on the dynamic execution required for open-ended work, which may be an effective complement to existing crowdsourcing process modeling.

On the other hand, quality control is not highlighted in the task models, such as by worker voting. Here, we do not analyze the output quality of different approaches in the evaluations. This is because the core of this paper is to explore whether CrowdModeller can provide the necessary support for the dynamic execution required by open-ended crowdsourcing work compared with other process modeling approaches. Therefore, in order to simplify the task models, we deliberately ignore the quality control. In fact, we believe that quality control is an important aspect of crowdsourcing research. We are planning to add appropriate quality control mechanisms (e.g., worker voting or machine algorithm [1]) to the task models to explore the differences between CrowdModeller and more different approaches.

Furthermore, we believe that the dynamic execution addressed in this paper is just one aspect of complex crowdsourcing processes. Indeed, resource dependencies and exception handling (or contingencies) are also important aspects needing to adapt to complex crowdsourcing processes [3, 4, 21, 25]. As a kind of resource dependencies, reasonable task allocation concerns assigning tasks to workers with appropriate skills. According to different factors (e.g., worker skills, demographics, compensation, and availability), many studies propose adaptive algorithms or skill models to achieve better task quality [1, 32–35]. Similarly, when considering exception handling, such as worker exit and dismissal, some researchers try to establish flexible organizational management mechanisms, such as Flash Team [25, 36], to adapt to these changing requirements.

How might CrowdModeller further improve its abilities to support complex crowdsourcing processes? We may draw on mature theory and technology of business process management (BPM) [20]. In fact, BPM is closely related to organizational behavior and process modeling. It is often used to optimize human resources, task allocation, and role-based team building to improve organization efficiency. With this in mind, CrowdModeller may enhance adaptability by utilizing the concepts of resource management and skill/role-based task allocation in BPM, which will be one of the directions of our follow-up research.

## 6. Conclusions

In this paper, we propose a crowdsourcing modeling approach for complex and open-ended work. It integrates task model composition, instance tree, and message communication modes to handle task creation, collaboration, and data exchange. Furthermore, we provide an automated execution framework called CrowdModeller, using graphical interfaces to define and monitor the crowdsourcing processes in real time. Finally, through two evaluations, we show that CrowdModeller can effectively support the dynamic execution of complex crowdsourcing processes. More specifically, the first evaluation, as a benchmark study based on article writing, shows that CrowdModeller can visualize the design, execution, and management of the whole crowdsourcing process, supporting the complex task modeling, collaboration, and data exchange requirements for dynamic execution. The second evaluation analyzes the difference between our approach and the state of the art for crowdsourcing process modeling. The results show that compared with the single fixed crowdsourcing model which tends to static control flow, CrowdModeller can coordinate multiple task models to match changing requirements, allowing flexible creation of task instances. Moreover, CrowdModeller provides user-friendly modeling tools and data display interfaces based on process modeling, which enables users to quickly design, implement, and manage crowdsourcing processes. This low-code user-friendly framework is conducive to reducing the programming workload and lowering the threshold of crowdsourcing network research.

In the future, we are planning to draw on the theory and technology of BPM to enhance the ability of CrowdModeller in terms of resource dependencies and exception handling. In addition, we are interested in exploring more crowdsourcing scenarios to further improve CrowdModeller.

## Figures and Tables

**Figure 13:**
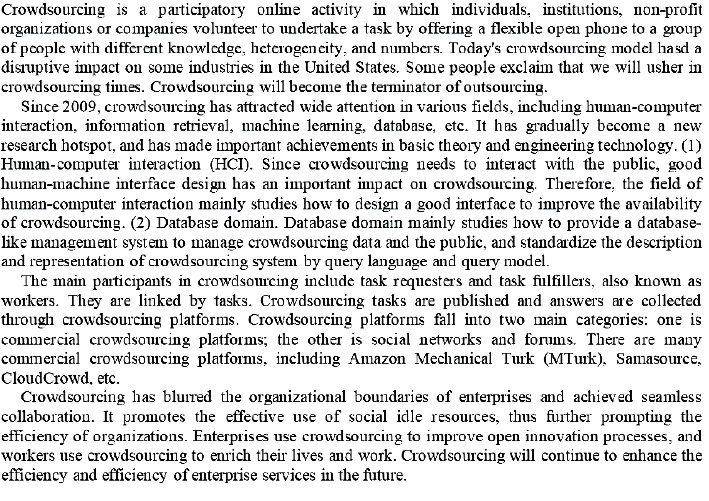
The experimental result of the article writing case based on CrowdModeller (round 1).

**Figure 14:**
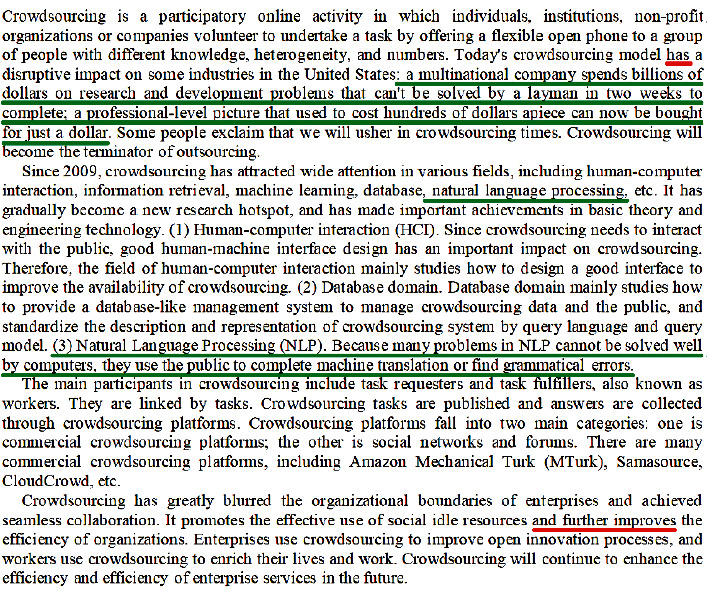
The experimental result of the article writing case based on CrowdModeller (round 2).

**Table 1:** An overview of existing approaches from multiple dimensions.

Dimensions	Approaches
Process definition language	JavaScript [5, 6], Dog [7], Scala [8], Python [9–11], custom modeling [12–16], BPEL [17], BPMN [18, 24]
Control flow mechanism	Iterative [5–7, 11, 12] [14–18, 24], recursive [9, 10], parallel [7–9, 11, 13–18, 24] approach
Data management	Data flow [5–18, 24]
Process development	Text + script [5–8, 10, 11], task template + script [9], editor [10], custom model + configuration [12–16], extended standard model + configuration [17, 18, 24]
Process execution	Static control flow [5–7, 9, 11–18, 24], algorithms based on budget or price [8, 10]

**Table 2:** Main statistics of the comparative evaluation. The final outputs are shown in the appendix.

Item	CrowdModeller	Service4Crowd
Task model	2, namely, 1 AVTM and 1 SAM	1
Task instances created by model (round 1)	6, namely, 1 AVTM task and 5 SAM tasks	1
Task instances created by model (round 2)	3, creating 3 new SAM tasks	0
Microtasks performed by workers (round 1)	14	8
Microtasks performed by workers (round 2)	10	7
Task management	Tree structure, that is, an instance tree supporting flexible task decomposition and any number of tasks	Linear structure, that is, a linear stream with fixed task decomposition and task quantity

## Data Availability

The source code of the framework has been uploaded to GitHub (https://github.com/xthHub/RenWFMS).

## Appendix

The experimental results of the article writing case are shown in Figures 13 and 14.
